# Meiotic segregation and interchromosomal effect in the sperm of a double translocation carrier: a case report

**DOI:** 10.1186/1755-8166-2-24

**Published:** 2009-12-01

**Authors:** Maria S Juchniuk de Vozzi, Silvio A Santos, Ciro S Pereira, Juliana F Cuzzi, Lucimar AF Laureano, José G Franco Jr, Lucia Martelli

**Affiliations:** 1 Department of Genetics, School of Medicine of Ribeirão Preto, University of São Paulo, Ribeirão Preto, SP, Brazil; 2 Genesis Genetics Brasil, Rua Mato Grosso, 306 cjt 506, 01239-040, São Paulo, SP, Brazil; 3 Center for Human Reproduction Prof Franco Junior, Av Prof João Fiusa, 689, 14025-310, Ribeirão Preto, SP, Brazil

## Abstract

**Background:**

Infertility is a natural mechanism of selection intended to prevent the delivery of a child with malformations or mental retardation. Male infertility is closely related to chromosomal abnormalities. This study was focused on the analysis of meiotic segregation involving a Robertsonian translocation, 45,XY,der(13;13) [56]/45,XY,der(13;14) [44] and the evaluation of possible interchromosomal effects.

**Results:**

Hybridisation with LSI 13q14 and subtelomere 14q probes and WCP13 SpectrumGreen and WCP14 SpectrumOrange probes showed a high proportion of unbalanced gametes, corresponding to 71.2% of the spermatozoa. The disomic frequencies of the sexual chromosomes and chromosome 18 of the patient were higher (5.28% and 2.55%, respectively) than those of the control (0.6% and 0.59%, respectively).

**Conclusion:**

Meiotic segregation studies in sperm are an important tool for genetic counselling of chromosomal aberrations, allowing for a prediction of the risks and consequent implications for the reproductive life. The patient with this rare translocation exhibited meiotic segregation fidelity, and a high rate of unbalanced gametes with disomic spermatozoa.

## Background

Infertility is a natural selective mechanism that affects one in six couples [1] that is intended to prevent the delivery of a child with congenital anomalies or mental retardation. Pregnancy can be difficult to achieve when previous attempts have been associated with recurrent abortions or implantation failures. Several studies investigating the different causes of infertility have included cytogenetic investigations to better understand the meiotic process [2-5]. Meiosis is a complex process and is monitored by different checkpoints, which are essential for proper cell division. Male and female gametes present different secondary responses to meiotic alterations. When an alteration occurs during spermatogenesis, meiosis halts, and apoptosis begins. On the other hand, if a problem occurs during oogenesis, meiosis continues to completion, thus generating aneuploid gametes. This fact could explain why the same chromosomal rearrangement causes male but not female infertility [6]. However, aneuploid gametes could still be generated during spermatogenesis if the meiotic checkpoints fail [7,8].

Male infertility is closely related to somatic chromosomal abnormalities. Many studies have demonstrated that chromosomal aberrations that cause meiotic interruption can lead to oligozoospermia or azoospermia, which produce abnormal gametes and lead to infertility [9].

The genetic causes of male infertility include chromosomal (aneusomies, translocations, Y microdeletions, inversions), genetic, mitochondrial and genomic imprinting factors. The most common abnormalities are gonosomal aneuploidies and Robertsonian translocations [10]. Aneuploidies can be caused by meiotic segregation errors, non-disjunction due to recombination defects, paternal age effects and kinetochore and microtubule alterations. In contrast, structural abnormalities require DNA breakage as a prerequisite for the formation of rearrangements in other chromosomes. During meiotic recombination, DNA breakage can increase the susceptibility for the loss or gain of genetic material in a chromosomal region [11]. Patients with Robertsonian translocations can produce 3.4-40% abnormal spermatozoa [12-16], while patients with reciprocal translocations have 47.5 - 81% abnormal germ cells [17-20].

Distinct combinations of Robertsonian translocations have been described for the five acrocentric chromosomes, with the 13;14 and 14;21 translocations being the most frequent. Although the Robertsonian translocation carrier is phenotypically normal, the abnormality contributes to genetic imbalances in the sibship, causing fetal losses, mental retardation, multiple congenital anomalies, uniparental disomy and infertility [21].

The first studies of meiotic segregation used heterologous *in vitro *fecundation [22,23]; later studies used fluorescent *in situ *hybridisation (FISH) techniques. The studies on meiotic segregation of chromosomes in the sperm of Robertsonian translocation males find a majority of normal or balanced spermatozoa for the chromosomes related to the translocation (mean 85.42%; range 60-96.60%) [24].

The influence of translocated chromosomes on the synapses and disjunction of other chromosomes is called an interchromosomal effect (ICE) [25]. ICE has been described in several chromosomal rearrangements. Chromosomal analysis of sperm in infertile males have shown high variability in chromosomal segregation behaviours during meiosis [26,27]. This variability could be associated with the multifactorial aetiology of male infertility, but in some cases, the combination of low sperm quality, chromosomal rearrangement and aneuploidies could affect meiotic synapses. It has been suggested that both the fluctuation in disomy and degree of semen parameter abnormalities are influenced by the chromosomes involved in the rearrangement. Different reports demonstrated an increased frequency of X and Y aneuploidies in patients with structural rearrangements involving autosomes [28,29,16,24]. Roux *et al*. (2005) [24] suggested that ICE could be detected in the sperm of Robertsonian translocation carriers, but this result could not be generalised.

This study analysed the meiotic segregation in a double Robertsonian translocation carrier with karyotype 45,XY,der(13;13)/45,XY,der(13;14) and the possible interchromosomal effects in the sperm.

## Results

**Figure 1:**
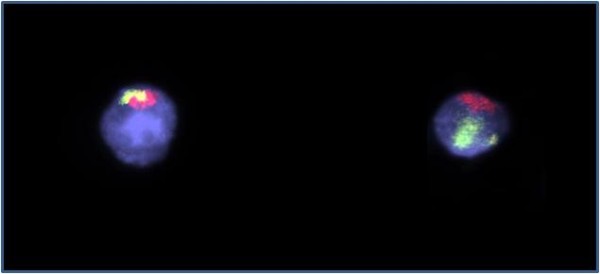
**A nucleus with the 13;14 translocation and normal sperm with one red signal (WCP 13) and one green signal (WCP 14)**.

**Figure 2:**
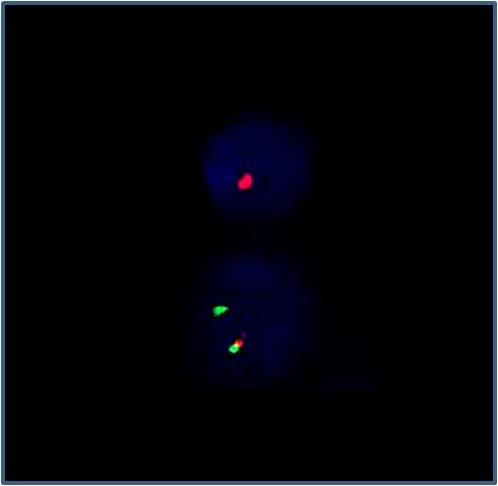
**FISH in spermatozoa using LSI 13 (green) and subtelomere 14 (red) probes**. One red signal and 13 nulisomic; one red signal and disomic 13 nucleus.

A total of 1831 patient spermatozoa were included in the meiotic segregation analysis of the chromosomes involved in the translocation. A FISH analysis using WCP probes for the chromosomes 13 (red) and 14 (green) was performed in 820 gametes (figure 1). The 13 LSI probe (green) and subtelomere 14 (red) analysis were performed in 1101 sperm (figure 2). The results are summarised in Tables 1 and 2. The hybridisation efficiency was 95% for LSI probes and 85% for WCP probes.

**Table 1:** The analysis of meiotic segregation of the patient using WCP probes.

Chromosome constitution	Signals	N	Rate
13/14 or der(13;13)	OG	307	37.43%
der(13;14)	OUG	248	30.21%
13/der(13;14) or der(13;13)	OOG	26	3.17%
14	G	181	22.1%
14/der(13;14)	OGG	36	4.4%
13	O	22	27%
		820	100%

O = orange; G = green; OUG = orange and green joined; OOG = two orange and one green; OGG = two green and one orange; OG = one orange and one green.

**Table 2:** The analysis of meiotic segregation of the patient using LSI probes.

Chromosome constitution	Signals	N	Rate
13/14 or der(13;14)	GO	291	28.78%
13/der(13;14) or der(13;13)/14	GGO	265	26.21%
14	O	321	31.75%
14/der(13;14)	GOO	48	4.75%
13	G	47	4.65%
Others	GG, OO, GGGO, GOOO	39	3.86%
		1011	100%

G = green; O = orange.

We also investigated the interchromosomal effects through a segregation analysis of chromosomes X, Y and 18. We detected 49.32% of sperm with one red signal, corresponding to the X chromosome, and 45.28% of sperm with one signal from the Y chromosome. Two signals (one red and one green) were observed in 0.78% of the gametes. We also observed two red signals (XX) in 0.28%, two green signals (YY) in 0.21% and disomy for chromosome 18 in 2.42% of the gametes.

The control analysis detected 49.1% of the gametes with one red signal (X), 49.3% with one green signal (Y), 0.25% with (XY), 0.16% with (XX) and 0.11% with (YY) disomic signals. The frequency of chromosome 18 disomy was 0.58% and diploidy was detected in 0.57% of cells.

## Discussion

Meiotic segregation analysis can predict gamete behaviour in patients with chromosomal abnormalities and can be useful for genetic counselling purposes. This study provides accurate information for the understanding of meiotic segregation in male gametes in this particular Robertsonian translocation carrier.

In the literature, a rare type of mosaicism has been described. Jumping translocations are events in which the same chromosomal segment is translocated to different chromosome sites in different cell lines [30]. Jumping translocations have mainly been described in haematological malignancies, but there also have been rare observations of a constitutional condition. This could explain the origin of the patient mosaicism, considering that the parental karyotypes were normal. Iwarsson et al., 2009 described a phenotypically normal male who presented a 45,XY,rob(13;13)(q10;q10)/45,XY,dic(13;15)(p11.2;p12) karyotype and oligoasthenoteratozoospermia, which is closely related to infertility. Another hypothesis for explaining this karyotype is chimerism, but further genetic informations would be necessary.

**Figure 3:**
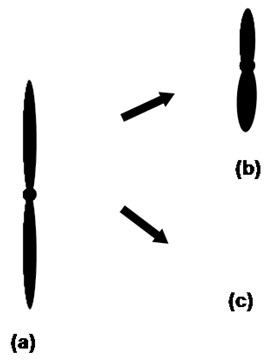
**The meiotic segregation of translocation t(13;13)**. a) A derivative chromosome, b) a disomic gamete and c) a nullisomic gamete.

We compared the segregation modes of the patient sperm with the segregation described in non-mosaic Robertsonian translocations because there have been no reports in the literature concerning this particular type of meiotic segregation. Only one similar patient was published by Iwarsson *et al*. [31]. Most of the meiotic segregation studies in sperm with Robertsonian translocations have concluded that the alternate mode of segregation is more frequent [29,24,4], indicating similar meiotic behaviour in different patients [13]. In our study, the balanced and normal gamete rates of alternate segregation were higher, in agreement with the literature [29,24,4] for the t(13;14) cellular line. The chromosome complement t(13;13) (Figure 3) increased the rate of unbalanced gametes and increased the risk of trisomy 13 in the descendant because it does not produce balanced gametes. Meiotic interruption, which leads to 3:0 chromosomal segregation, could explain the observed rates in the sperm of the Robertsonian translocation carrier. Another explanation could be meiotic checkpoint intervention. Any factor able to modify anaphase (such as chromosomal rearrangement or the loss of microtubule tension) could interrupt the cell cycle [32].

In the meiotic segregation of the patient with 13;13 and 13;15 mosaicism, the rate of abnormal spermatozoa was 45.2%, which is lower than our study (72.22%). This could be explained by the genome of the involved chromosomes.

Comparing the meiotic segregation analysis in sperm and the frequency of abnormal embryos, Escudero *et al*. [13] concluded that meiotic segregation analysis are a good predictor for the rate of abnormal embryos. They concluded that there is a reasonable chance of conceiving when abnormal sperm rates are less than 65%. Our results have identified 71.22% abnormal gametes, including the alternate and adjacent modes of segregation. Consequently, our findings suggest a poor prognosis of conception, with an increased risk for fetal loss or trisomies in successful conceptions.

ICE remains controversial in the literature, although some publications have indicated its relevance [16]. Anton *et al*. [29] observed high sex chromosome aneuploidy rates (0.68%) in the sperm of Robertsonian translocation carriers compared with controls (0.37%) and insignificant rates for other chromosomes. In our study, we observed high levels of disomy for the sex chromosomes, in agreement with the literature [33,28]. Our results showed even higher rates than previously reported [24,34]. The checkpoints involved in meiosis could have failed, producing aneuploid gametes. Luciani *et al*. [35] described a non-random association between the trivalent and the sex chromosomes in prophase I in male mice. A similar study in mice with Robertsonian translocations also described an association between the trivalent and the sex chromosomes. Other groups described the association between the trivalent and sex chromosomes as the affinity between the inactive XY body and the asynaptic regions of the multivalent. This region is covered by the variant histone γ-H2AX, which increased this association and led to transcriptional inactivation [36,37]. The interference of the heterosynapses, the mechanism that rescues the gamete when anaphase is interrupted, could explain the increase of XY disomy. The interference of the trivalent in meiosis might affect the segregation of chromosome 18; its disomy frequency was increased when compared with the control (p < 0.01). Another study also reported an increased frequency of chromosome 18 disomy [34]. Non-disjunction and the absence of recombination could also explain these rates [38].

When the meiotic segregation of a translocation is studied, the chromosomes involved in the translocation, as well as the genetic condition of each patient, must be considered. Meiosis is a very complex process; any change in the configuration and position of the chromosomes will increase the likelihood of meiotic arrest or errors.

## Conclusion

We conclude that the study of meiotic segregation in the sperm of Robertsonian translocation carriers provides information about chromosomal imbalances and the increased risk of producing children with genetic syndromes. In our patient, we demonstrated accurate meiotic segregation, high rates of unbalanced gametes and disomic sexual germ cells in this rare translocation. Genetic counselling is required to explain the poor reproductive outcome to the patient. The analysis of the segregation of chromosomes involved in the translocation and ICE can generate a more personalised risk assessment for the reproductive outcome of patients with structural chromosome aberrations that can be useful in genetic counselling.

## Methods

Two sperm samples from a patient with the 45,XY,der(13;13)(q10;q10) [56]/45,XY,der(13;14)(q10;q10) [44] karyotype were collected in sterile collectors. The phenotypically normal patient was 30 years old and presented oligozoospermia. At the same time, two sperm samples from a control (30 years old, fertile man without abnormalities, normal male karyotype and normal spermiogram) were also collected. The affected patient and the control were informed about the study, and written informed consent, approved by the Ethical committee, was obtained.

The samples were washed in 0.01 M Tris/0.9% NaCl solution. Slides were made with 5 μl of the sample and stored at -20°C.

### FISH technique

Two sets of probes, WCP (whole chromosome painting) for chromosome 13 (cat. N° LPP13R, Cytocell, UK) and for chromosome 14 (cat N° LPP14G, Cytocell, UK) and locus specific sequence (LSI) for chromosomes 13, LSI 13 (13q14) SpectrumGreen (cat N° 32-192018, VYSIS Inc., USA) and TelVysion 14q SpectrumOrange (cat. N° 33-260014, VYSIS Inc., USA) were used for the meiotic segregation studies. CEP X (DXZ1), Y (DYZ1) sat III (cat N° 30-161050, VYSIS Inc., USA) and CEP 18 (D18Z1) SpectrumOrange (cat N° 32-130018) and SpectrumGreen (cat N° 32-132018) probes (VYSIS Inc., USA) were used for the ICE analysis.

The samples were decondensed in 10 mM DTT(dithiothreitol)/0.01 M Tris solution and LIS (lithium 3,5-diidosalicylate) solution at room temperature. They were dehydrated through an ethanol series and air-dried. Then they were immersed in 0.005% pepsin solution (Sigma, USA) for 10 minutes to eliminate the cytoplasm. The probes were prepared according to the manufacturer instructions. The slides were denatured in a water bath at 74°C. The probe mix was then applied to the slides, which were covered with a coverslip, sealed with rubber cement and hybridised overnight in a moist chamber at 37°C. The slides were washed in 50% formamide and 2× SSC solution, and DAPI (4',6-Diamino-2-phenylindole) was used for counterstaining. The results were analysed with an epifluorescence Zeiss microscope coupled with the Metasystem 2.1 software. Only morphologically intact sperm were assessed, according to standard assessment criteria. Overlapping sperm nuclei, disrupted nuclei or large nuclei with diffuse signals were not considered [39]. In the painting assays, nuclei with two signals of different colours clearly coupled one with the other, were considered as displaying a balanced chromosomal pattern [40].

### Data analysis

The statistical analysis was performed using the SAS system (2002-2003, SAS Institute Inc., Cary, NC) [41]. The data related to the analysis of the meiotic segregation of chromosomes X, Y and 18 were analysed by the Chi-Square test (χ^2^).

## Consent

The affected patient and the control were informed about the study, and written informed consent, approved by the Ethical committee, was obtained.

## Competing interests

The authors declare that they have no competing interests.

## Authors' contributions

MSJV was responsible for the project, methodology, results and discussion. SAS and LAFL provided cytogenetic support. CSP and JFC provided FISH support and methodology. FJ provided the clinical information and LM was the advisor and coordinator. All of the authors read and approved the final manuscript.
